# Sex differences in shoulder performance fatiguability are affected by arm position, dominance and muscle group

**DOI:** 10.1186/s12891-022-05232-w

**Published:** 2022-03-29

**Authors:** Cosmin Horobeanu, Samuel A. Pullinger, Julien Paulus, Cristian Savoia, Fui Yen Wong, Antoine Seurot, Jean L. Croisier, Benedicte Forthomme

**Affiliations:** 1 Aspetar, Qatar Orthopaedic and Sports Medicine Hospital, Doha, Qatar; 2 Sport Science Department, Inspire Institute of Sport, Vidyanagar, Dist., Bellary, 583275 India; 3 Département Des Sciences de La Motricité, Faculté de Médecine, Université de Liège, Liège, Belgium; 4 Research Institute for Sport and Exercise Sciences (RISES), Tom Reilly Building, Liverpool John Moores University, Liverpool, UK; 5 Academy of Defence Fitness, National Defence University of Malaysia, Kuala Lumpur, Malaysia; 6 Département Physiothérapie, Swiss Olympic Medical Center, Meyrin, Suisse

**Keywords:** Muscular performance, Fatigue resistance, Isokinetic, Shoulder rotator muscles, Interaction

## Abstract

**Background:**

Injury prevalence data, muscle strength, and fatiguability differ between males and females. In addition, arm spatial orientation affects muscle activation and strength of the shoulder muscles. Nevertheless, little research has been conducted in relation to the shoulder rotator muscles comparing men and women. Therefore, the main aim of of this study was to perform a comparative investigation between two arm spatial orientations (45° and 90° of abduction in the frontal plane) during a fatigue assessment of the internal rotator (IR) and external rotator (ER) shoulder muscles. Secondly, the interaction between sex and dominance with muscular performance was assessed.

**Methods:**

Forty healthy sedentary participants, 20 males and 20 females took part in this study. Participants performed a fatigue resistance protocol consisting of 30 consecutive maximal concentric contractions of the IR and ER shoulder muscles in a supine position at a speed of 180°/s. The upper limb was abducted to an angle of 45° or 90° in the frontal plane and each participant was tested on the dominant and nom-dominant side, counterbalanced in order of administration. Performance measures of Induced Fatigue (IF; %), Cumulated Performance (C.Perf; J) and Best Repetition (BR; J) were calculated and used for analysis. IF represents the % difference between the amount of work done over the last 3 and first 3 repetitions, BR represents the largest amount of work done during a single contraction, and C.Perf represents the total amount of work done during all repetitions.

**Results:**

Muscle group was the only factor to display significant variation when not considering other factors, with higher values for C.Perf (mean difference = 353.59 J, *P* < 0.0005), BR (mean difference = 14.21 J, *P* < 0.0005) and IF (mean difference = 3.65%, *P* = 0.0046). There was a significant difference between both angles, with higher values observed at 90° compared to 45° of abduction for C.Perf by ~ 7.5% (mean difference = 75 to 152 J) and ~ 10.8% (mean difference = 5.1 to 9.4 J) for BR in the ER, in males and females respectively (*P* < 0.0005). The dominant arm was significantly stronger than the non-dominant arm for C.Perf by 11.7% (mean difference = 111.58 J) for males and by 18% (mean difference = 82.77 J) for females in the ER at 45° abduction. At 90° abduction, only females were stronger in the dominant arm by 18.8% (mean difference = 88.17 J). Values for BR ranged from 9.2 to 21.8% depending on the abduction angle and sex of the athlete (mean difference = 2.44 – 4.85 J). Males were significantly stronger than females by 48.8 to 50.7% for values of C.Perf and BR in both the IR and ER (*P* < 0.0005). There was a significant difference between the ER and IR muscles, with significantly higher values observed for the IR in C.Perf (mean difference = 331.74 J) by 30.0% and in BR (mean difference = 13.31 J) by 26.64%.

**Discussion:**

Differences in shoulder performance fatiguability between sexes are affected by arm position, arm dominance and muscle groups. In agreement with the literature, performance values in males were approximately 50% higher than in females. However, the amount of IF was no different between both sexes. Based on findings in literature, it could be suggested that this is due to differences between males and females in motor control and/or coordination strategies during repetitive tasks. In addition, we also observed the IR muscles to be significantly stronger than the ER muscles. It has long been established in literature that these observations are due to the muscle-size differences between both muscle groups, where the IR muscles can produce a larger amount of force due to the larger cross-sectional area. Results of our study found similar ER:IR ratios compared to previous reports.

**Conclusion:**

Therefore, these findings are useful for clinicians when monitoring rehabilitation programs in sedentary individuals following shoulder injuries.

## Background

The use of isokinetic dynamometry has extensively been used by clinicians and scientists to assess the muscular performance of different muscle groups. Collected data can objectively be used for the diagnosis, the evaluation and the monitoring of specific rehabilitation and training programs [1–3]. Previous findings have established that muscular fatigue negatively affects human performance due to a reduction of the muscular force generating capacity that occurs during repetitive muscular contractions [4, 5]. It has been observed that fatigue in the shoulder rotator muscles has implications on upper limb activity [5, 6] and has been associated with sub-acromial impingement injury [7]. Injury prevalence data indicates that females are at higher risk than their male counterparts when it comes to musculoskeletal disorders in the shoulder area in a sedentary population [8, 9]. In addition, females have also shown greater fatigue resistance for a variety of isometric tasks [8–10]. The endurance time for fatiguing contractions has been found to be shorter for men in the elbow flexor muscles (118%) and the elbow extensors (11-min *vs.* 17-min) compared to women [11, 12]. Fatigue resistance time for the knee extensors was also found to be lower in males when compared to females (180-s *vs*. 252-s), while females were also able to complete more repetitions to exhaustion in elbow-flexion compared to males [13]. Differences in muscle mass and hormones between males and females are some of the factors which have previously been suggested as the cause of these observed differences [14]. Further, the type of assessment (e.g., isometric, isotonic or isokinetic), the number of contractions, the speed of contraction, the muscle group and the age of participants are other factors which have shown to affect findings [15].

Isokinetic assessments of the internal rotators (IR) and external rotators (ER) in the shoulder have previously been investigated to assess muscular performance [16–19]. The use of the isokinetic machine to asses the fatigue resistance of shoulder rotator muscles using different protocols has previously shown to establish very high reproducibility [20–22]. Differences between the IR and ER muscles have previously been observed, with the magnitude highly affected by muscle size [21, 23] and the angle of arm abduction [24]. Nevertheless, it is difficult for clinicians to compare findings between previous studies looking at shoulder rotator muscles due to large differences in methodological qualities. The variation between protocols used in the literature is large and very little research has been conducted to assess differences between males and females.

Current observations within the literature clearly establish that differences between testing positions greatly influence the properties of muscular strength [18, 24], highlighting the need for the fatigue assessment protocol to be reproducible [3, 21, 25]. Previous research has found that the optimal position for isokinetic assessment of the shoulder rotators muscles’ strength was in a supine position with the arm abducted in a frontal plane, at an angle of 45° or 90° [24]. A recent investigation conducted by Horobeanu et al. [21] established that lying in a supine position with the arm abducted in a frontal plane is a suitable and reliable option for assessing fatigue resistance in the shoulder rotators, irrespective of whether arm abduction amounts to 45° or 90°. However, a study conducted by Golebiewska et al. [26] found that an increase in the abduction angle in frontal plane from 45° to 90°, caused a decrease in torque values, thus stressing the importance for more research to be conducted within this area.

Previous findings have established that 30 consecutive maximal concentric contractions on the IR and ER shoulder muscles in a supine position at a speed of 180°/s, with the upper limb abducted to an angle of 45° or 90° in frontal plane is a reliable and valid protocol to assess fatigue resistance. It is currently unknown as to whether previously observed differences would still be present when assessing both males and females, in the dominant and non-dominant limbs. Although differences have previously been established between males and females in a number of different studies, findings are highly dependent on the body part (e.g., ankle, arms, lower back) and the type of muscle contractions (e.g., isometric or isotonic) utilised [27, 28]. Therefore, due to the diversity of assessment protocols used it is very difficult for clinicians to establish whether assessment of fatigue resistance in shoulder rotator muscles would yield similar results as other body limbs. In addition, previous findings related to limb dominance effects on muscular performance as a result of fatigue differ and are not well established [29, 30]. To our knowledge there is no work investigating the influence of arm spatial orientation (in the frontal plane) on shoulder rotators performance during a fatigue resistance assessment while considering differences between sexes and arm-dominance. We consider this type of evaluation to be of interest for scientists involved in overhead sport disciplines and clinicians engaged in treating different shoulder pathologies. Assessments of shoulder muscular performance and aspects of functional dynamic stability, by incorporating fatigue, is useful for clinicians and can help with the set-up and monitoring of specific rehabilitation programs and provide a clearer understanding in relation to injury in a sedentary population.

Therefore, the main aim of this study was to perform a comparative investigation of muscular performance, between two arm spatial orientations (45° and 90° of abduction in frontal plane), during an isokinetic fatigue resistance assessment of the shoulder rotator muscles. The second aim was to assess the possible interactions of sex and dominance taking into account the muscular performance of both IR and ER.

## Materials and methods

### Design

A cross-sectional design was used to compare muscular performance in the shoulder rotator muscles following a fatigue resistance assessment between males and females.

### Participants

Forty sedentary young adults, of which 20 males (mean ± SD: age 23.6 ± 2.3 yrs, height 1.79 ± 0.06 m and body mass 72.5 ± 10.7 kg) and 20 females (mean ± SD: age 22.1 ± 1.8 yrs, height 1.66 ± 0.06 m and body mass 58.7 ± 7.4 kg) volunteered for this study. Inclusion criteria required the participants to meet sedentary classification by not undertaking any regular physical activity and/or meeting the physical activity guidelines set by the World Health Organisation (WHO). None of the participants were practicing upper limb (recreational or professional) sport or involved in regular upper arm activities. None had a history of upper extremity bone fractures and/or a history of musculoskeletal abnormality; and none of the participants were receiving any pharmacological treatment during this study. Only female participants meeting the following criteria were included within the research: a) a consistent, “normal” menstrual cycle during the last 3 months; b) no use of any hormonal contraceptive use during the last 3 months; c) menstruating over the last 12 months. All tests were performed during the mid-luteal phase of the menstrual cycle, conforming to similar studies in the literature [31].

All participants were free to live a “normal life” between testing sessions. They were told to refrain from drinking alcoholic or caffeinated beverages and from heavy training or exertion 48 h prior to each experiment. Verbal explanation of the experimental procedure was provided to everyone; this included the aims of the study, the possible risks associated with participation and the experimental procedures to be utilised and all questions were answered. Individuals then provided written, informed consent before participating in the study. The experimental procedures were approved by the Human Ethics Committee at the Faculté de Médecine, Université de Liège. The study was conducted in accordance with the ethical standards of the journal and complied with the Declaration of Helsinki. All the participants used in the study were right-hand dominant, except for 2 female and 4 male participants. Hand-dominance was self-declared and corresponded to the hand they use to write with. In addition, all participants also provided informed consent for publication of identifying information/images in an online open-access publication.

### Procedure

All sessions took place under standard laboratory conditions. Before taking part in the main experiment, each participant completed one familiarisation session. This session ensured that participants were fully familiarised with the experimental conditions required for the study. During the familiarisation session, participants were required to perform as many repetitions as possible until they felt comfortable performing maximal concentric contractions on the dominant and non-dominant shoulder at speeds of 120°/s and 180°/s. Following the familiarisation process, all participants then completed two identical experimental sessions which took place 10 days apart to minimise any learning effect and to exclude any muscle soreness. Both sessions were conducted at the same time-of-day to reduce potential influence of circadian rhythmicity [32].

**Fig. 1:**
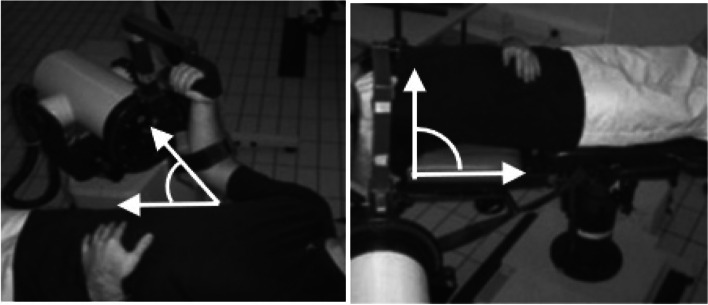
Protocol set up displaying the upper limb abducted in the frontal plane in a supine position at an angle of 45° or 90°, respectively

Prior to the experimental session, all participants undertook a standardised warm-up consisting of 2 sets of 20 consecutive repetitions concentric contractions on the dominant IR and ER shoulder muscles in a supine position using an elastic band of heavy strength (blue) attached to a fixed point allowing the elbow to be bent at 90° and be maintained close to the body. Once the standardised warm-up was completed, the participants were asked to sit in a comfortable position on the dynamometer (Biodex Medical Systems, Shirley, New York). The position of the participant in the chair was standardized in accordance with the guidelines set by the manufacturers and took into consideration any adjustment required by the individual (established during the familiarisation session). These guidelines have been published previously [18]. Participants were then asked to undertake a task-specific warm-up consisting of 10 gradual repetitions at 120°/s and 3 sub-maximal repetitions at 180°/s, after which a 3-min rest period was provided. Once completed they performed the fatigue resistance protocol, which consisted of 30 consecutive maximal concentric contractions of the IR and ER shoulder muscles in a supine position at a speed of 180°/s at maximal intensity. The upper limb was abducted to an angle of either 45° or 90° in the frontal plane and each participant was tested on the dominant and non-dominant side, with these counterbalanced in order of administration (Fig. 1). The range of movement was pre-set on every occasion to mimic the testing conditions; 70° for ER and 50° for IR. All participants confirmed to be comfortable and pain free during both sessions. Standardised strong verbal encouragement and live feedback was given during all sessions.

### Outcome measures

The work (in Joule; J) during each repetition (*x*) was computed and used to calculate the performance for following parameters individually: 1) Induced fatigue (IF); 2) Cumulated Performance (C.Perf); and 3) Best repetition (BR). The following formulas have been used for calculations:

1. IF (%) – represents the difference between the amount of work done over the last 3 and first 3 repetitions:
  $$IF=\left(\left(\frac{average work done during last 3 reps}{average work done during first 3 reps}\right)\mathrm{x }100\right)-100$$
2. C.Perf (J) – represents the total amount of work done during all repetitions:
  $$C.Perf={\sum }_{30}^{1}x$$
3. BR (J) – represent the largest amount of work done during a single contraction:
  $$BR=\mathrm{max }x$$

### Statistical analyses

All data were analyzed using JASP statistical analysis software and R analytics. All data were checked for normality using the Shapiro–Wilk test. A repeated measures ANOVA with paired group comparison was used to compare the 3 parameters (*n* = 160) for: 1) differences between arm position (45° vs 90°); 2) differences between arm dominance (dominant *vs*. non-dominant); and 3) muscle group differences (IR *vs*. ER). In addition, a repeated measures ANOVA with independent group comparison was used to compare the 3 parameters (*n* = 20) between sexes (male *vs.* female). The results are presented as the mean ± the standard deviation throughout the text unless otherwise stated. Following convention, the alpha level of significance was set at 5% where values of *P* < 0.05 have been referred to as “significant”. Values of “0.000” given by the statistics package are shown here as *P* < 0.0005 [33]. The Bonferroni correction was used for the repeated measures ANOVA to increase the significance.

## Results

Table 1 provides the mean ± SD values and statistical significance for all performance variables and each factor (arm position, dominance and muscle group) without consideration of sexes. The IR muscles showed statistical significance with higher values for C.Perf (mean difference = 353.59 J, *P* < 0.0005), BR (mean difference = 14.21 J, *P* < 0.0005) and IF (mean difference = 3.65%, *P* = 0.0046) compared to the ER muscles. Arm position and dominance did not display any significant differences (*P* > 0.05).

**Table 1:** Mean ± SD values and statistical significance for IF (%), C.Perf (J) and BR (J) for arm position (45° vs. 90°), arm dominance (Dom vs. Non-Dom) and muscle group (ER vs. IR) without consideration of sexes

	**Arm position**			**Arm dominance**			**Muscle Group**		
	**45°**	**90°**	***P*-value**	**Dominant**	**Non-dominant**	***P*-value**	**IR**	**ER**	***P*-value**
**IF (%)**	-42.60 ± 11.34	-42.81 ± 11.42	*P* = 0.870	-41.91 ± 10.51	-43.58 ± 12.18	*P* = 0.200	-40.91 ± 11.60	-44.56 ± 10.94	***P* = 0.046**
**C.Perf (J)**	750.44 ± 441.51	809.39 ± 411.76	*P* = 0.390	982.36 ± 448.19	933.58 ± 448.19	*P* = 0.350	1136.06 ± 477.59	782.47 ± 320.81	***P* < 0.0005**
**BR (J)**	34.26 ± 19.38	37.89 ± 18.77	*P* = 0.320	44.16 ± 18.96	42.33 ± 19.45	*P* = 0.400	50.43 ± 20.28	36.22 ± 14.91	***P* < 0.0005**

Table 2 shows the mean ± SD values for all variables and all factors considering their interactions. There was a significant difference between both angles, with higher values observed at 90° of abduction compared to 45° abduction for C. Perf in males by 7.5% (mean difference = 152.3 J) and in females by 7.6% (mean difference 74.94 J) in the ER. Values for BR were also significantly higher at 90° of abduction compared to 45° abduction for C. Perf in males by 10.2% (mean difference = 9.40 J) and females by 11.3% (mean difference 5.11 J) in the ER (*P* < 0.0005). There was no significant difference for IF between the angles (*P* = 0.43) in the ER or any of the assessed parameters in the IR (*P* > 0.05).

**Table 2:** Mean ± SD values for IF (%), C.Perf (J) and BR (J) for differences between muscle groups (ER *vs.* IR), arm positions (45**°** *vs.* 90**°**), and dominance (dominant *vs.* non-dominant) in males and females considering interactions

			**45°**		**90°**	
			**IR**	**ER**	**IR**	**ER**
**Females**	**Non-dom**	**IF (%)**	-40.11 ± 16.55^b^	-46.71 ± 12.15	-40.35 ± 14.35	-43.82 ± 16.63
		**C.Perf (J)**	722.81 ± 160.59^b^	455.17 ± 129.00	745.09 ± 184.15^b^	489.94 ± 113.80^a^
		**BR (J)**	32.82 ± 6.81^b^	21.38 ± 4.88	33.85 ± 7.49^b^	23.23 ± 5.18^a^
	**Dom**	**IF (%)**	-39.82 ± 13.03	-41.27 ± 9.55	-39.60 ± 16.35	-42.51 ± 9.89
		**C.Perf (J)**	688.18 ± 238.93^b^	537.94 ± 126.92^d^	709.93 ± 239.08^b^	578.11 ± 116.10^a,d^
		**BR (J)**	31.65 ± 11.15^b^	23.82 ± 4.94^d^	32.84 ± 11.20^b^	27.08 ± 5.56^a,d^
**Males**	**Non-dom**	**IF (%)**	-41.80 ± 13.67	-45.77 ± 7.86	-39.09 ± 10.68^b^	-46.90 ± 8.29
		**C.Perf (J)**	1435.28 ± 319.71^b,c^	951.69 ± 201.40^c^	1494.37 ± 443.87^b,c^	1059.56 ± 228.89^a,c^
		**BR (J)**	63.72 ± 20.15^b,c^	43.71 ± 9.72^c^	65.07 ± 19.73^b,c^	49.86 ± 11.32^c^
	**Dom**	**IF (%)**	-39.60 ± 11.62	-41.62 ± 11.31	-38.71 ± 9.06^b^	-46.69 ± 8.55^b^
		**C.Perf (J)**	1532.84 ± 352.7^b,c^	1063.27 ± 189.16^c,d^	1532.80 ± 357.44^b,c^	1107.70 ± 189.80^a,c^
		**BR (J)**	67.31 ± 16.27^b,c^	48.13 ± 9.76^c,d^	66.07 ± 14.01^b,c^	51.38 ± 9.61^a,c^

^a^highlights significant differences with higher values at 90° angle compared to 45° angle (*P* < 0.05)

^b^highlights significant higher values in the IR compared to the ER (*P* < 0.05)

^c^highlights males significantly stronger than females (*P* < 0.05)

^d^highlights dominant arm significantly stronger than non-dominant arm (*P* < 0.05)

There was a significant difference between arm-dominance, with the dominant arm significantly stronger than the non-dominant arm for C.Perf in males by 11.7% (mean difference = 111.58 J) in the ER at 45° abduction. In females, the dominant arm was significantly stronger by 18% (mean difference = 82.77 J) at 45° abduction and 18.8% (mean difference = 88.17 J) at 90° abduction in the ER. Values for BR were also significantly higher for males by 9.2% (mean difference = 4.42 J) and for females by 11.4% (mean difference = 2.44 J) at 45° abduction. Females also had significantly higher values for BR at 90° abduction by 21.8% (mean difference = 4.85 J) in the ER (*P* < 0.0005). There was no significant difference for any of the other values assessed (*P* > 0.05).

Males were significantly stronger than their female counterparts for all values of C.Perf and BR, in both arms, at both angles and for both shoulder muscles. Males were significantly stronger by 50.7% for C.Perf (mean difference = 2121.06 J) and 50.7% for BR (mean difference = 98.02 J) in the ER compared to the females at both angles. Males were also significantly stronger for C.Perf by 48.8% (mean difference = 2728.47 J) and BR by 50.0% (mean difference = 131.01 J) in the IR compared to the females at both angles (*P* < 0.0005). There was no significant difference for IF between males and females for dominance, muscle group or angle (*P* > 0.05).

There was a significant difference in muscle group between the ER and IR, with higher values observed for the IR compared to ER across all factors. Values for the IR are significantly higher than the ER by 30.0% in C.Perf (mean difference 331.74 J) and 26.64% in BR (mean difference = 13.31 J). There was no significant difference for IF between muscle groups (*P* = 0.62).

## Discussion

The main findings of this study established major differences between males and females in shoulder performance fatiguability with arm position, dominance and muscle group playing an important role. Previous findings have established that the arm spatial orientation affects muscle activation and strength of the shoulder muscles [24, 34]. In agreement, our results observed significant differences in C.Perf and BR between the angle of abduction and muscle group in both males and females. However, induced fatigue did not establish any differences between any of the comparisons. Nevertheless, it is well recognised that overhead athletes are at a higher risk of shoulder related injuries with the ligaments around the shoulder being weakened due to the overload, repetitive stress and fatigue [35, 36]. This is due to a reduction in the subacromial strength, as a result of the higher angles of abduction [7], especially when the rotator cuff muscles are in a fatigued state [37]. We found no differences in induced fatigue, but it must be noted that our participants did not undergo a repetitive stress/overload over an extended time-period.

The most notable finding of our study was that all measures related to performance were statistically different between the IR and ER shoulder muscles in both males and females. It was found that the IR muscles were more fatigue resistant (IF: 8.19%), able to perform higher amounts of work over 30 repetitions (C.Perf: 45%) and able to develop more work during a single repetition (BR: 39.23%) compared to the ER muscles. Previous findings have also found IR muscles to be significantly stronger than the ER muscles [23, 38]. This is due to the muscle-size differences between both muscle groups, where the IR muscles can produce a larger amount of force due to the larger cross-sectional area. In addition, the IR muscles have a larger lever arm than the ER muscles, meaning more force can be produced [20]. Further, there are differences from a biomechanical perspective between the IR and ER shoulder muscles in relation to size and volume which further accentuates the significant differences found in favour of the IR muscles. The results of our study also found similar results to previous reports related to ER:IR ratios which seems to be between ~ 1:2 and 9:10 in pain-free sedentary individuals [23, 39, 40].

Another finding of the current study was that no differences in performance (IF, C.Perf, BR) were present between supine positions when the arm is abducted to a 90° angle *vs.* a 45° angle, when only considering the arm position. In addition, no differences were observed in arm-dominance with values between the dominant and non-dominant arm not statistically different. Therefore, considering previous findings in relation to changes in IR and ER muscle performance at different abduction angles, it was important to further investigate the possible interaction of angle and dominance. Our data suggests that increasing the abduction angle to 90° from 45° positively impacts the work of ER on BR in both sexes and the dominant and non-dominant arm, while no differences were observed for the IR. These results contradict the findings of Golebiewska et al. [26] who observed a decrease in muscular strength from 45° to 90° of abduction in the frontal plane for both muscle groups. Our study assessed induced fatigue, work done over all repetitions and the best repetition, while Golebiewska et al. [26] looked at peak torque. Peak torque looks at only one point of the movement, the highest one on the angular curve, while the work represents the whole area under the curve [41]. This helps explain potential differences between these studies. Peak torque is not necessarily the etalon of all other torques developed through the entire range of movement, and only has a consistent occurrence between certain degrees of movement.

When assessing fatigue resistance, the IR were deemed to be less “fatigued” than the ER after 30 repetitions in both the dominant arm (-38.71% *vs.* -46.69%) and the non-dominant arm (-39.09% *vs*. -46.94%) for males at the 90° position while the females were less fatigued on the non-dominant side at 45° (-40.11% *vs*. -46.71%) only. Similar profiles have previously been found in the literature with the IR displaying more fatigue resistance than the ER in a study performed by Ellenbecker & Roetert, [42] when looking at young tennis players. Differences in fatigue rates between the IR and the ER have a clinical relevance. The ER function as a humeral head stabilizer [36] especially for the athletic population where it has been shown to alter performance [6]. In addition, it has also been shown to be a potential shoulder injury risk factor. Differences in shoulder position affect the muscle activation around the shoulder and its rotational strength [35, 43]. Studies on a larger population and from variate age groups are necessary to explore their influences and possible implications for different rehabilitation programs of non-athletic populations.

Further, it has been established that males are stronger compared to females when comparing different muscles groups [44–47]. Sex differences have previously been found for knee antagonist muscles, quadriceps and hamstrings, with males performing between 25 to 40% more work compared to their female counterparts [45, 46] during maximal concentric reciprocal contractions. Females have also previously been shown to have between 37 and 68% of the muscle strength observed in males in several measures of upper body strength. Significant differences have previously been established in shoulder strength with differences approximately 50% higher in males in both the upper shoulder (deltoid and trapezius) and the lower shoulder (Latissimus) muscles [48]. Our findings also observed shoulder rotator muscles in males to be significantly stronger than females (*P* < 0.05) by ~ 50%, for both arms and at both angles of shoulder abduction. The amount of difference between sexes should be viewed from the perspective of raw data, without normalisation to body weight. The choice of comparing raw values has been agreed in order to make possible comparisons with other findings in the literature.

Finally, our findings failed to establish differences between males and females on fatigue resistance of the shoulder rotator muscles, showing both sexes having a similar reduction in performance, ranging from 38 to 47%, after 30 reciprocal concentric contractions. Present results are in line with findings established by Senefeld et al. [47], who reported no significant differences between both sexes. However, our findings are in contradiction with results observed by Avin et al. [44], who showed females were more fatigue resistant than men. It must be noted that in their study the participants were tested for their isometric sustained capabilities at elbow level, as opposed to the shoulder muscles. We support the affirmation of Hunter et al. [28] that the sex differences regarding fatigue resistance is task specific because different neuromuscular sites are stressed when the requirements of the task alters, and the stress on these sites can differ for men and women. Task variables that can alter the sex difference in fatigue resistance include but are not limited to the type, intensity and speed of contraction, the muscle group assessed, and the environmental conditions. There seems to be a considerable lack of understanding in the literature on fatigability and its associated physiological mechanisms [49] as to why sex differences are present. It is known that females have better muscular endurance than men in several parts of the body, such as lower back, thighs and arms. The contraction intensity also determines whether differences are observed between both sexes. A longer time to exhaustion has been foind in women at lower intensities during maximal voluntary capacity [12], but when performing dynamic work, these differences are no longer present [50]. It is believed that differences are due to males and females using different motor control/coordination strategies during repetitive tasks to preserve functional aspects of task performance [49]. Our findings agree with some of the previous findings, but we also believe that it is important to gain a better understanding on the impact of menstrual cycle phases on the effect of study design and potential outcomes. It has been shown that different phases show different fluctuations in performance levels [50, 51]. In order to gain a better understanding, monitoring females during different stages of the menstrual cycle will provide a clearer picture. Nevertheless, differences between males and females are apparent, but explanations for these differences remain unanswered and more research is required in order to supplement the current literature.

## Conclusion

The results of this study showed that when assessing muscular performance of the shoulder rotators in a sedentary population shoulder abduction at 45° or 90° in the frontal plane does not influence muscular fatigue. Therefore, either of these two positions can be considered appropriate to assess functional dynamic stability in sedentary individuals. When considering shoulder muscle strength, a change in the arm position, from 45° to 90° of abduction significantly affects the ER muscles, in favour of the 90° angle, while the IR muscles are not affected. Although it is well established that males are stronger that females in performance measures related to work done (C.Perf and BR), there seems to be no fatigue differences between sexes on any of the upper limbs. These findings are useful for clinicians working with sedentary people when monitoring the progression and outcome of their rehabilitation programs after certain shoulder injuries and provides us a better understanding of future shoulder joint assessments clinical interpretation.

## Data Availability

The datasets used and/or analysed during the current study are included in this published article and more detailed data is available from the corresponding author on reasonable request.

## Abbreviations

ER: External Rotator muscles
IR: Internal Rotator muscles
IF: Induced Fatigue
C.Perf: Cumulated Performance
BR: Best Repetition
Dom: Dominant arm
Non-Dom: Non-Dominant arm
ES: Effect Size
